# Multidimensional plasticity of natural killer cells in tumours

**DOI:** 10.1038/s41419-025-08361-x

**Published:** 2025-12-22

**Authors:** Xinya Yang, Zhaoyang Song, Ligang Chen, Qingge Jia, Mingyang Li

**Affiliations:** 1 Department of Pathology, Xijing Hospital and School of Basic Medicine, Fourth Military Medical University, Xi’an, China; 2 Department of Clinical Medicine, School of Medicine, Yan’an University, Yan’an, China; 3 Department of Neurosurgery, General Hospital of Northern Theater Command, Shenyang, China; 4 Department of Reproductive Medicine, Xi’an International Medical Center Hospital, Northwest University, Xi’an, China

**Keywords:** Immunosurveillance, Cancer immunotherapy

## Abstract

Natural killer (NK) cells exhibit remarkable adaptability within the tumour microenvironment (TME), where dynamic shifts in phenotype, function and metabolism govern their dual roles in antitumour immunity and tumour immune evasion. In the TME, NK cells undergo receptor remodelling, which is characterised by upregulated inhibitory signals and suppressed activating receptors, leading to the formation of dysfunctional subsets, such as exhausted TIM-3⁺ NK cells or tissue-resident CD49a⁺ populations. Immunosuppressive factors within the TME drive a transition from cytotoxic activity to regulatory or senescent-like states, impairing tumour surveillance. Metabolic reprogramming further compromises NK cell effector functions, as nutrient deprivation and metabolic byproducts disrupt energy pathways and suppress immune responses. Therapeutic strategies targeting this plasticity include engineered natural killer (NK) cells with enhanced specificity, metabolic restoration approaches and microenvironment-modulating interventions. However, challenges persist because of TME heterogeneity and persistent dysfunctional states. Understanding these adaptive mechanisms provides a framework for developing NK cell-based therapies that leverage plasticity to counteract tumour resistance.

## FACTS

- Natural killer cells exhibit remarkable adaptability within the tumour microenvironment, where dynamic shifts in phenotype, function and metabolism govern their dual roles in anti-tumour immunity and tumour immune evasion.
- Therapeutic strategies targeting this plasticity include engineered NK cells with enhanced specificity, metabolic restoration approaches and microenvironment-modulating interventions.
- Understanding these adaptive mechanisms provides a framework for developing NK cell-based therapies that leverage plasticity to counteract tumour resistance.

## Introduction

Natural killer (NK) cells, which are derived from bone marrow haematopoietic stem cells, play a central role as effector cells in the innate immune system. Studies have shown that under physiological conditions, there are three types of NK cells in human bone marrow: NK1, NK2 and NK3. These three primary NK cell populations can be divided into six subgroups and the NK2 cell population also shows a tumour-specific trend [1–3]. NK cells exhibit a rapid response mechanism and rely on germline-encoded receptors such as NKG2D⁺ and DNAM-1⁺ to effectively identify and eliminate both malignant tumour cells and virus-infected cells [4–6]. Moreover, different subsets of NK cells are present in the tumour microenvironment (TME), whose expression of different markers differs, resulting in functional differences, as listed in Table 1 [7–12]. The cytotoxic potential of NK cells is precisely regulated by a dynamic balance between activating receptors (such as NKp30, NKG2D⁺, NKp44 and NKp46) and inhibitory checkpoint molecules (for instance, KIRs, NKG2A and TIGIT). This regulatory equilibrium allows NK cells to discriminate between healthy cells and stressed target cells, as comprehensively summarised in [13, 14].

**Table 1:** NK cell subpopulation.

Subpopulation	Markers	Function	Cytolytic factors	Reference
Exhausted NK cell	TIM-3⁺, PD-1, LAG-3	cytotoxicity was lost and IFN-γ secretion decreased	IFN-γ	[7]
Immunosuppressive NK cell	CD39, CD73, A2AR	Adenosine is generated and T/NK cell activity is inhibited	IFN-γ, CCL5, XCL1	[8]
Tissue-resident NK cell	CD49a⁺, CD103, CXCR6	Long-term resident tissues secrete VEGF to promote angiogenesis	VEGF、SDF-1、IP-10	[9, 10]
Adaptive NK cell	CD2, CD57, CD85j	Memory-like reactions, enhancing NK cell responsiveness	IFN-γ, TNF-α	[11]
(ILC1)-Like NK cell	CD49a+CD49b+	Secrete pro-angiogenic factors and be pro-tumorigenic	(PDGF)-AB	[12]
dNK-Like Cells	CD56brightCD16-/dim and express CD9 and/or CD49a.	Decrease proinflammatory and cytolytic capacities and produce pro-angiogenic factors.	pro-angiogenic factors	[12]

Nevertheless, the immunosuppressive TME disrupts this delicate balance through multiple interrelated mechanisms. First, the TME deprives NK cells of metabolic energy through the accumulation of waste products such as lactate, which directly impairs bioenergetic homoeostasis. Simultaneously, energetic reprogramming occurs in NK cells, involving alterations in genetic regulation that induce a functional state characterised by metabolic exhaustion, epigenetic silencing and phenotype remodelling [15, 16]. This synergistic effect of the TME leads to the exhaustion of NK cells, driving multidimensional plasticity in their phenotype, function and metabolic profile.

Recent research has shed light on the dynamic adaptability of NK cells within the TME. For example, sex-specific energetic regulation has been reported: NK cell-specific knockout of UTX significantly inhibited lung metastasis of melanoma in male mice but had no such effect in female mice. These findings are consistent with the documented mechanism by which TME-mediated epigenetic silencing impairs NK cytotoxicity, including hypermethylation of the NKG2D⁺ promoter and histone deacetylation at the DNAM-1⁺ locus [17]. In non-small cell lung cancer (NSCLC), the TME is enriched with myeloid-derived suppressor cells (MDSCs) that exhibit a CD39⁺CD73⁺ phenotype —a feature not observed in infiltrating NK cells, which primarily undergo functional exhaustion via other pathways (e.g. upregulation of the exhaustion marker TIM-3) rather than CD39/CD73 expression [18]. These MDSCs establish an immunosuppressive signalling axis called the ‘adenosine-A2AR axis’ to directly inhibit T cell function, while also impairing NK cell activity indirectly. Specifically, the CD39 protein on MDSCs first hydrolyses extracellular ATP (an energy-providing molecule for cells) into AMP; subsequently, the CD73 protein further converts AMP into adenosine. The adenosine accumulated in the TME then binds to the adenosine A2A receptor (A2AR) expressed on the surface of both T cells and NK cells [19].

For T cells, adenosine-A2AR binding directly suppresses their proliferation and cytotoxicity, weakening their ability to kill tumour cells. For NK cells, this binding does not disrupt oxidative phosphorylation (OXPHOS) or glycolytic pathways— mechanisms that lack support in current NSCLC-focused studies—but instead blocks their maturation process: it reduces the proportion of mature cytotoxic NK cell subsets (e.g. CD27⁻CD11b⁺ in murine models, CD56dimCD16⁺ in humans) and impairs their secretion of interferon-γ (IFN-γ) (a key cytokine that enhances anti-tumour immunity). Consequently, the NK cells show reduced responsiveness to IL-2 (a molecule that stimulates immune cell activation), diminished cytokine secretion and blunted anti-tumour cytotoxicity—ultimately reinforcing the immunosuppressive state of the TME [18, 19]. This metabolic–immune crosstalk establishes a positive feedback loop of immunosuppression, which is further amplified by hypoxic conditions and TGF-β signalling in the TME.

Additional evidence emphasises the role of soluble noninflammatory factors in NK cell polarisation. In sepsis models, activation of the IL-10/IDO axis promotes the differentiation of NK cells into a regulatory phenotype (CD56^bright^CD16^−^). Notably, IDO, which is highly expressed in most human tumours, is closely correlated with the loss of NK cell cytotoxicity, highlighting its significance as an immunosuppressive factor secreted by the TME [20, 21]. The spatial heterogeneity of NK cell subsets has been revealed through prognostic modelling in EBV-associated gastric cancer, differentiating between TIM-3⁺ exhausted subsets and CD49a⁺ tissue-resident subsets. Tissue-resident NK cells, which are adapted to specific TMEs, typically exhibit reduced cytotoxic function and variable production of inflammatory cytokines [22–24]. Collectively, these findings demonstrate that the TME impairs the antitumour function of NK cells through coordinated energetic, metabolic and phenotypic reprogramming.

In response to these mechanisms, emerging chemotherapeutic strategies adopt combination approaches. Engineered chimeric antigen receptor-NK (CAR-NK) cells, especially fourth-generation constructs incorporating IL-15 or logic-gated antigen recognition modules, increase cell persistence and target specificity. Metabolic interventions, including NAD⁺ supplementation to restore OXPHOS capacity and IDO inhibitors to counteract tryptophan depletion, aim to revitalise the epigenetic and functional responsiveness of NK cells. Environmental remodelling strategies, such as TGF-β siRNA carriers and oncolytic viruses (e.g. oHSV-CXCL10), seek to disrupt the immunosuppression network by modulating cytokine signalling and enhancing immune cell infiltration [17]. Despite these advancements, significant challenges remain, including the spatial heterogeneity of tumour lesions, host immune rejection of engineered cells and complex interactions with the extracellular matrix.

In conclusion, the complex interactions between NK cells and the TME necessitate the development of multidimensional therapeutic strategies. These approaches must integrate the molecular mechanisms of NK cell dysfunction with the unique constraints of the tumour environment, promoting the design of more effective and personalised chemotherapeutic interventions.

## Phenotype plasticity: dynamic receptor remodelling and subset heterogeneity

NK cell phenotypic plasticity is operationally defined as receptor network remodelling measurable by flow cytometry (e.g. CD49a⁺ tissue-resident subset enrichment) and epigenetic assays (e.g. NKG2D⁺ promoter hypermethylation). The phenotypic plasticity of NK cells within the TME involves a highly dynamic adaptive process meticulously choreographed by competitive receptor–ligand interactions and profound microenvironmental reprogramming. This biological manifestation originates from the delicate equilibrium maintained between activating receptors—such as NKG2D⁺ and DNAM-1⁺, which trigger cytotoxicity cascades—and inhibitory receptors such as killer immunoglobulin-like receptors (KIRs) and TIGIT. The latter recognises subsequently expressed major histocompatibility complex (MHC) class I molecules on healthy cells. When MHC class I engages with inhibitory KIRs, it transduces dominant negative signals that effectively suppress NK cell effector functions. This process establishes an evolutionarily conserved surveillance mechanism designed to eliminate MHC-I-deficient targets while safeguarding against autoimmunity [25–27]. Under normal physiological conditions, this MHC class I-dependent inhibitory signalling efficiently curtails NK cell activation. However, tumours frequently downregulate MHC class I expression as an immune evasion strategy. In theory, this should render them vulnerable to NK cell-mediated dialysis [14, 28]. Paradoxically, advanced tumours subvert this surveillance system by inducing compensatory mechanisms. These mechanisms give rise to heterogeneous NK cell populations with functionally distinct phenotypes, highlighting the intricate nature of tumour–immune interactions [29].

Central to this adaptive process is the bidirectional remodelling of receptor networks. In hepatocellular carcinoma (HCC), the CD155-TIGIT axis serves as a prime example of how tumours exploit inhibitory checkpoints. The high-affinity binding of TIGIT to CD155 inhibits NK cells through two mechanisms: by recruiting SHP-2 adenylyl cyclase to the cell surface to degrade the phosphorylated products of the PI3K/Akt pathway, thereby preventing the endocytosis cycle of NKG2D⁺ or by competing with CD155 for binding, thus preventing the activation of the receptor DNAM-1⁺ to prevent interaction and subsequent triggering of signal transduction. TIGIT, which has the highest binding affinity for CD155, interacts with overexpressed CD155 on tumour cells. This interaction delivers potent inhibitory signals that override concurrent activating receptor stimulation, such as that mediated by NKG2D⁺ [30]. Previous experiments have revealed a new mechanism in liver cancer in which the high expression of CD155 on platelet-adhered circulating tumour cells (CTCs) enables them to evade NK cell death. Blocking the binding of CD155 to TIGIT can effectively increase the clearance of circulating CTCs by NK cells [31]. Clinical investigations have demonstrated that elevated TIGIT expression on NK cells is associated with functional exhaustion and an unfavourable tumour prognosis [32]. As a result, TIGIT blockade has emerged as a promising therapeutic approach to reinstate NK cell cytotoxicity [33]. Simultaneously, CD155 functions as a ligand for the activating receptor DNAM-1⁺ on NK cells [14, 17, 30]. In the TME, DNA damage responses lead to the release of soluble CD155 (sCD155). This sCD155 competes for DNAM-1⁺ binding, promotes receptor degradation and downregulates activating signalling [34]. Mechanistically, TIGIT further disrupts DNAM-1⁺homogenisation, preventing its interaction with membrane-bound CD155. Consequently, this abrogates the costimulatory signals crucial for NK cell activation [35]. Lysosomal-mediated communication adds another layer of regulation. HCC-derived exosomal circHRF1 transfers miRNAs to NK cells, posttranscriptionally regulating PD-1 expression. This exacerbates the exhaustion phenotype beyond imbalances in the receptor expression profile. Maelstrom components further reinforce this suppressive environment. Adipose-derived mesenchymal stem cells (MSCs) secrete TGF-β, whose transcription downregulates NKG2D⁺ and DNAM-1⁺ by reducing DAP10 mRNA and protein levels. This functionally disconnects NK cells from stress ligand recognition [36].

Energetic reprogramming stabilises these dysfunctional phenotypes through chromatin-level modifications that establish heritable gene expression patterns. Recent studies have revealed a critical mechanism of immune escape involving the proteolytic shedding of NKG2D ligands and altered membrane topology. To clarify the biological basis of this mechanism, NKG2D (NK cell lectin-like receptor subfamily D) acts as a key activating receptor on NK cells. The ligands of NKG2D are mainly divided into two families: MHC class I-related molecules A/B (MICA/B) and UL16-binding proteins (ULBPs, e.g. ULBP1-6). Under physiological conditions, these ligands are weakly expressed on healthy cells; however, in tumour cells, stress signals such as DNA damage, oxidative stress or viral infection can significantly upregulate their expression. When NKG2D ligands bind to NKG2D on NK cells, they activate downstream cytotoxic signalling pathways (e.g. PI3K/Akt and MAPK), thereby promoting the release of granzyme B and perforin, as well as the secretion of IFN-γ, which together mediate the killing of tumour cells. Notably, tumour cells have evolved a ‘ligand shedding’ strategy to escape NKG2D-mediated immune surveillance. This process proceeds in two sequential steps: first, endoplasmic reticulum protein 5 mediates the cleavage of disulphide bonds in the α3 domain of MICA/B; then, metalloproteases (e.g. ADAM10/17 and MMP14) further cleave the modified MICA/B, releasing soluble MICA/B (sMICA/B) into the TME. Secreted sMICA/B can bind to NKG2D on the surface of NK cells, inducing receptor internalisation and degradation, which ultimately inhibits NK cell activity. Clinically, elevated serum levels of sMICA/B are observed in patients with melanoma and NSCLC and this elevation is associated with poor survival outcomes [37]. Compared with peripheral or liver-resident NK cells, tumour-infiltrating NK cells exhibit significantly fewer membrane protrusions, as visualised by transmission and scanning electron microscopy. This defect arises from reduced sphingomyelin levels in NK cell membranes, driven by tumour-induced dysregulation of serine metabolism. Mechanistically, serine depletion in the TME impairs SM biosynthesis, leading to disrupted membrane protrusion formation and reduced immune synapse formation [38]. In ovarian cancer, hypermethylation of CpG islands in the NKG2D⁺ promoter region induces long-term transcriptional silencing. In melanoma, histone deacetylase (HDAC)-mediated chromatin compaction at the DNAM-1⁺ locus impedes transcriptional activation [39–41]. These epigenetic changes create a long-lasting ‘memory’ of dysfunction. They persist even in the absence of continuous TME signals and synergise with surface receptor modulation to enforce sustained NK cell paralysis.

The spatiotemporal heterogeneity within the TME drives subset specialisation through niche-specific cues. In EBV-associated gastric cancer, the hypoxic tumour core is enriched in TIM-3⁺ exhausted NK cells, characterised by impaired IFN-γ production and reduced cytotoxic potential. Comparative analysis in colorectal cancer reveals distinct CD16 expression profiles. Compared with their peripheral blood counterparts, tumour-infiltrating NK cells display significantly lower CD16 levels, while adjacent normal tissues exhibit intermediate expression. This highlights the phenotypic divergence between tissue-resident and circulating NK cell populations. In contrast, cardiovascular NK cells retain the CD16⁺ cytotoxic phenotype. They mediate antibody-dependent cellular cytotoxicity (ADCC) through immunoreceptor tyrosine-based activation motif (ITAM) signalling in the CD3ζ chain, which is coupled with high-affinity IgE receptors [38, 42, 43]. This compartmentalisation is exemplified in glioblastoma. In contrast to peripheral CD56dimCD16+ cells, which maintain cytotoxic function, tumour-infiltrating CD56^bright^CD16^−^ NK cells secrete VEGF and IL-10 to promote pathogenesis and immunosuppression [44]. Hypoxia-inducible factor 1α (HIF-1α) and TGF-β gradients drive this specialisation. HIF-1α stabilises CD73⁺ expression to facilitate adenine-mediated immunosuppression, while TGF-β induces differentiation into tissue-resident CD49a⁺⁺CD103⁺ NK cells at the expense of migratory cytotoxic subsets [12]. Notably, this plasticity is environmentally dependent. Ectopic Eomes expression in innate lymphoid cells (ILC1s) can convert them into NK-like cells with restored cytotoxicity in murine models. This challenges the traditional concept of irreversible lineage commitment in tissue-resident populations [45]. Clinical correlations include chronic hepatitis B-induced chemokine receptor reprogramming (CCR1 downregulation/CXCR6 regulation) in circulating NK cells, which impairs their homing to infected hepatocytes. Additionally, extranodal NK/T-cell lymphoma drives PD-1/TIM-3⁺coexpression on NK cells, which is associated with shortened progression-free survival [46, 47].

**Fig. 1:**
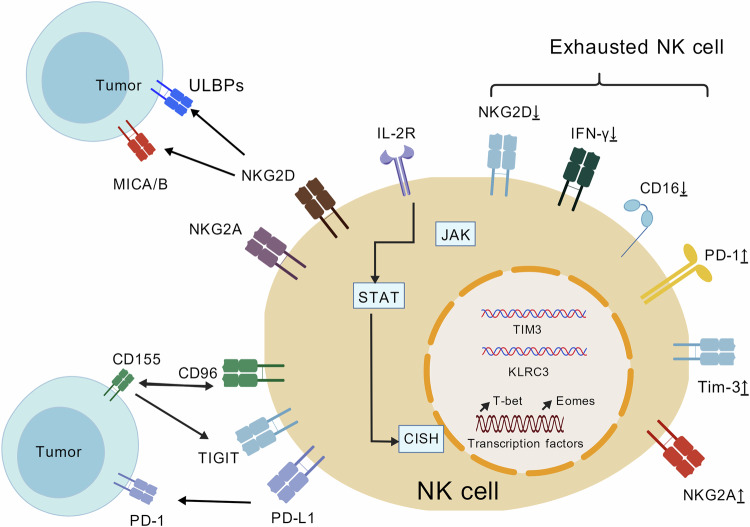
Multidimensional regulatory mechanisms of NK cell phenotypic plasticity in the TME. NKG2D (NK cell lectin-like receptor subfamily D): Activating receptor that recognises stress ligands including MICA, MICB and ULBPs and enhances the killing activity of NK cells against tumours; NKG2A (NK cell lectin-like receptor subfamily A): inhibitory receptors that bind HLA-E (such as CD155) to transmit inhibitory signals and mediate tumour immune escape; CD96 (T-cell activation inhibitory receptor): immune checkpoint molecule that binds to CD155 to inhibit NK cytotoxicity; CD155 (poliovirus receptor): tumour ligand that bidirectionally regulates NK cell function (activated by DNAM-1⁺ and inhibited by CD96/TIGIT); IL-2R (interleukin-2 receptor): mediates IL-2 signalling and drives NK cell proliferation and effector function through the JAK-STAT pathway (JAK, STAT5); CISH (cytokine-induced SH2 protein): negatively regulates IL-2 signalling and degrades STAT5 to prevent overactivation; PD-L1 (programmed death ligand 1): an immune checkpoint ligand that binds to PD-1 on NK cells to inhibit their function and mark the exhaustion state; TIM-3⁺ (T cellular immunoglobulin mucin 3): a depletion marker that binds to galectin-9 to inhibit NK cell activity. This figure was created with BioRender.com.

The convergence of receptor dynamics and subset specialisation constitutes a ‘double-hit’ immunosuppression mechanism. Transcriptional silencing of activating receptors (e.g. NKp30 promoter hypermethylation) works in tandem with checkpoint expression (e.g. TIGIT and PD-1) to disrupt both target recognition and effector function. Transcript analyses underscore the microenvironmental regulation of receptor plasticity. Compared with peripheral blood cells, bone marrow-derived NK cells exhibit distinct inhibitory receptor profiles (e.g. KIR2DL1 predominance), as depicted in Fig. 1. Figure 1 further delineates the multidimensional regulatory network underlying this receptor plasticity, highlighting three key regulatory axes: first, the activating–inhibitory receptor balance: NKG2E (an activating receptor) recognises stress ligands such as MICA/B to enhance NK cell killing, while NKG2A (an inhibitory receptor) binds HLA-E (e.g. CD155) to transmit suppressive signals—this balance is disrupted in the TME, where tumours upregulate NKG2A ligands to silence NK cells [30]; second, CD155-mediated bidirectional regulation: CD155 on tumour cells acts as a ‘molecular switch’: it activates NK cells via DNAM-1⁺ binding but inhibits them via CD96/TIGIT interaction. In HCC, platelet-adhered CTCs overexpress CD155, which preferentially binds TIGIT on NK cells to block NKG2D⁺ endocytosis and PI3K/Akt signalling, enabling CTC escape [31]. Third, cytokine‒transcription factor crosstalk occurs: IL-2R mediates JAK‒STAT5 signalling to promote NK cell proliferation, but this pathway is countered by CISH (a negative regulator that degrades STAT5). In the TME, reduced IL-2 availability and increased CISH expression synergise to weaken activating signals, while upregulated PD-L1 and TIM-3⁺ (exhaustion markers) further lock NK cells into dysfunction [29]. Collectively, Fig. 1 illustrates how TME-driven perturbations in these axes converge to remodel NK cell receptors, laying the foundation for subset heterogeneity (e.g. TIM-3⁺ exhausted cells and CD49a⁺ tissue-resident cells), as discussed earlier. These findings reflect the plasticity of the NK cell phenotype in the TME and these cell surface proteins affect the different states of NK cell activation and inhibition. Overcoming these layered regulatory mechanisms requires integrated therapeutic strategies. These strategies should target both cell-intrinsic plasticity—through epigenetic modulators and receptor-engineered CAR-NK cells—and extrinsic TME signals—such as TGF-β inhibitors and hypoxia-targeting agents—to restore NK cell anti-immunosuppression competence.

## Functional plasticity: from cytotoxicity to carcinogenicity

**Fig. 2:**
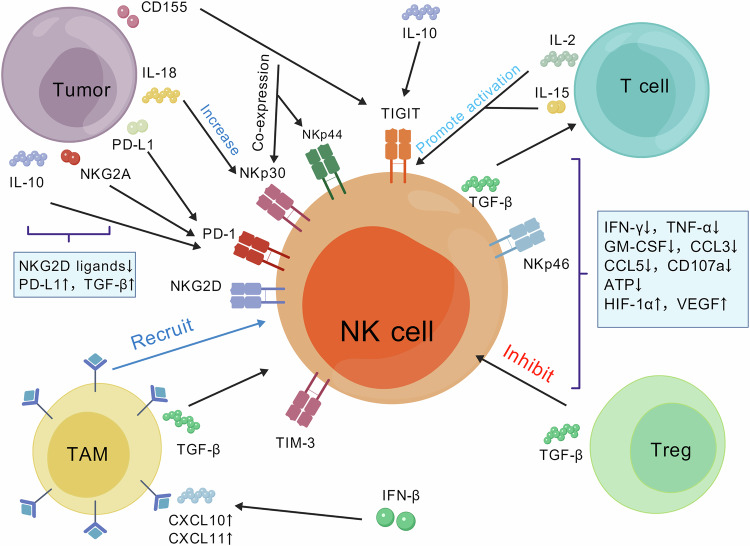
NK cell functional state molecular network map: activation and inhibition signalling pathways and cytokine profiles. Proinflammatory cytokines (IFN-γ, TNF-α and GM-CSF) and chemokines (CCL3, CCL5 and CXCL10/11) drive immune activation, whereas CD107a is involved in NK cell degranulation. NK cell surface receptors (NKp46, NKp44, TIM) and inhibitory checkpoints (PD-1, NKG2A) reflect functional modulation. Immunosuppressive factors (IL-10, TGF-β and PD-L1) and hypoxia-related markers (HIF-1α and VEGF) highlight tumour-driven immune evasion. This figure was created with BioRender.com.

Functional plasticity—quantified by cytotoxic assays (e.g. reductions in granzyme B secretion) and cytokine profiling (e.g. upregulation of IL-10 expression)—reflects the hierarchical molecular reprogramming that drives NK cells from cytotoxic effectors to regulatory states under TME stress. This intricate phenotype transition is meticulously orchestrated by hierarchical molecular reprogramming spanning receptor signalling, energetic modification and metabolic adaptation. At the heart of this process lies the dynamic disequilibrium between activating and inhibitory receptor networks (Fig. 2) [48, 49]. The molecular circuits that govern NK cell functional shifts are shown in Fig. 2, with clear delineation of activating cues, inhibitory signals and functional readouts: activating pathways: Proinflammatory cytokines (IL-2, IL-15 and IL-18) and activating receptors (NKp46, NKp44 and NKG2D) drive the secretion of pro-tumouricidal factors (IFN-γ, TNF-α and GM-CSF) and degranulation (marked by CD107a). For example, IL-15 sustains NK cell survival and cytotoxicity via mTOR activation, whereas the chemokines CXCL10 and CXCL11 recruit NK cells to tumour margins [50, 51]. Inhibitory perturbations: the TME disrupts these pathways through multiple mechanisms: (1) tumour-derived TGF-β and IL-10 downregulate activating receptors (NKG2D, NKp30) and reduce cytokine secretion (IFN-γ↓, CCL3↓/CCL5↓); (2) immune checkpoint ligands (PD-L1) and stromal cells (Treg, TAM) reinforce suppression—Tregs secrete TGF-β to block granzyme B expression, whereas TAMs increase adenosine signalling to inhibit NK cell activation [52, 53]; and (3) hypoxia (marked by HIF-1α↑) induces VEGF secretion, shifting NK cells towards a pro-angiogenic state rather than a cytotoxic state. Functional readouts: the chart links these signals to measurable functional changes: exhausted NK cells show reduced CD107a (degranulation) and ATP levels, whereas regulatory NK cells upregulate IL-10 and lose antitumour cytokine production. This finding aligns with our earlier observation in triple-negative breast cancer, where TGF-β induces a TOMM20-low mitochondrial phenotype that impairs granzyme B exocytosis [54]. This network map (Fig. 2) explains why NK cells transition from cytotoxic effectors to immunosuppressive regulators in the TME: inhibitory signals overwhelm activating cues, leading to coordinated loss of cytotoxic function and increased pro-tumour activity. There is complex crosstalk between NK cells and various TMEs. NK cells and TMEs sometimes cooperate with each other, but more often, they fight against each other, thus eliciting different antitumour responses [54]. Moreover, mutual interference occurs between NK cells and other immune cells in the TME [55]. Previous studies have shown that NK cells under IL-12 or IL-2 conditions can initiate DC maturation of Th1 cells that produce IFN-γ. NK cells under IL-18 conditions induce Th1 polarisation only when they are cocultured with DCs and T cells, whereas the release of IL-2 by T cells promotes the production of IFN-γ. When NK cells are under IL-4 conditions, they produce nonpolarized T cells that release only low levels of IL-2 [56]. Treg cells in the TME can also interfere with NK cells because of their sensitivity to immunosuppressive agents [57]. Previous studies have shown that Tregs directly inhibit NK cell responses through the activity of transforming growth factor (TGF)-β, thereby affecting their antitumour activity [52].

Prolonged exposure to tumour-derived factors promotes receptor desensitisation through the transcriptional downregulation of pivotal activating receptors such as NKG2D, DNAM-1 and NKp30. Intriguingly, this occurs despite the intact preservation of the core effector machinery, effectively muting the cytotoxic potential and pushing NK cells into a paradoxical state of functional tolerance. This context-dependent plasticity manifests diversely in different biological scenarios. For instance, Yishen-Jiangu pills alleviate osteoarthritis inflammation by suppressing synovial NK cell activation via inhibition of the ERK/NK-1 receptor pathway. Conversely, in breast cancer models, the epigenetic silencing of cytotoxicity receptors mediated by TGF-β/SMAD3 is achieved through histone deacetylation at the TBX21 locus, underscoring the tissue-specific nature of these adaptive mechanisms [58].

TGF-β orchestrates NK cell functional plasticity through a hierarchical triad of mechanisms. In vitro studies have shown that TGF-β treatment blocks IL-15-induced mTOR phosphorylation of S6 kinase (S6K) and 4EBP1, reducing NK cell metabolic activity (e.g. glucose uptake and OXPHOS) by 40–60%. This inhibition is comparable to the effect of the mechanistic target of rapamycin complex 1 (mTORC1) inhibitor rapamycin, as both TGF-β and rapamycin reduce NK cell proliferation, granzyme B expression and cytotoxicity against tumour cells. Mechanistically, TGF-β signalling via SMAD2/3 represses mTOR activity, uncoupling IL-15-mediated metabolic reprogramming and impairing effector function. Additionally, the mRNA endonuclease Regnase-1 acts as a negative regulator of IFN-γ production. Its deletion relieves the transcriptional repression of OCT2 (Pou2f2) and IkBζ (Nfkbiz), which form a complex with NF-κB to bind a distal enhancer (DI + 55) 55 kb downstream of the IFN-γ promoter. This interaction increases IFN-γ transcription by 4.6-fold, as confirmed by ChIP-seq, which revealed increased H3K27 acetylation at the DI + 55 locus in Reg1ΔNK-NK cells [50].

The first aspect is epigenetic silencing mediated by TGF-β. SMAD3 recruits HDACs to TBX21/PRF1 promoters, inducing chromatin compaction that silences T-bet and granzyme B. Lactate in the TME further inhibits HDAC activity, promoting NKG2D promoter methylation—a type of metabolic‒epigenetic cross-talk that exacerbates receptor downregulation [59, 60]. The second aspect is receptor signalling network perturbation. In HCC, TGF-β1 upregulates CD96, disrupting the CD226-CD96-TIGIT axis. This receptor perturbation, combined with NKG2D downregulation, forms a dual blockade of activation signals, reducing NK cell cytotoxicity [61]. The last aspect is mitochondrial metabolic dysfunction. In triple-negative breast cancer, TGF-β1 induces a TOMM20-low phenotype, disrupting mitochondrial protein import and OXPHOS. The resulting ATP depletion impairs granzyme B exocytosis, directly compromising cytotoxicity. TGF-β-coordinated immunosuppressive pathways play a role in this process. TGF-β‘s triad of mechanisms involving TGF-β synergises with canonical immunosuppression pathways. For example, activation of the IL-10/IDO axis polarises NK cells to a CD56brightCD16− regulatory phenotype. Moreover, paradoxical IFN-γ-JAK1/STAT1 signalling upregulates PD-L1 expression on NK cells, establishing a self-perpetuating loop of immunosuppression through the inhibition of PD-1^+^ T cells. The interaction between PD-1 and PD-L1 results in the recruitment of protein tyrosine phosphatase 2 (SHP-2), which in turn downregulates PI3K/Akt signalling to maintain immune tolerance [62, 63].

Spatial cues within the TME further determine the functional polarisation of NK cells. A reduction in membrane protrusions impairs the formation of lytic immunological synapses, as demonstrated by reduced tight junction formation and F-actin polarisation in tumour-infiltrating NK cells. Coculture experiments revealed that compared with >90% of peripheral NK cells, only 21–50% of intratumoural NK cells bind to tumour cells. This defect is correlated with decreased granzyme B polarisation and cytotoxicity, as measured by flow cytometry and real-time cell index assays [38]. Single-cell sequencing of EBV+ gastric cancer reveals spatial segregation of the following NK subsets: IFN-γ-high cells at vascularises margins and PD-L1+ cells in hypoxic cores. This correlates with local lactate gradients (>10 mM in cores), directly linking metabolic stress to phenotypic polarisation [64]. Adipose-derived MSCs secrete TGF-β and prostaglandin E2 (PGE2). These factors downregulate the production of perforin and granzyme B, regulate PD-L1 expression, disrupt IL-15-mediated survival signalling in NK cells and paradoxically increase NKG2D⁺ surface expression [50].

Lactate accumulation, a characteristic feature of TME acidosis, induces metabolic paralysis through dual mechanisms. Tumour acidosis (pH 6.2–6.5) impairs cytokine signalling by reducing the binding of IL-2 to IL-2Rα (EC₅₀ ↑2.3-fold at pH 6.5). This results from histidine protonation at the IL-2:IL-2Rα interface, disrupting receptor dimerisation. In B16 melanoma, acidosis suppresses IL-2-induced STAT5 phosphorylation by 40–60%, a defect reversed by bicarbonate neutralisation [65].

In CRLM, tumour-derived lactate (11.5 ± 2.8 mmol/L, pH 6.6) induces liver-resident NK cell apoptosis via mitochondrial ROS accumulation, a process rescued by MitoTempo [66]. Additionally, the immune checkpoint receptor TIM-3 plays a pivotal role in NK cell dysfunction via ligand-mediated signalling. Among its four ligands, galectin-9 has emerged as the most potent suppressor of NK cell cytotoxicity and proliferation. In head and neck squamous cell carcinoma (HNSCC), galectin-9 binding to TIM-3 impairs NK cell-mediated killing by disrupting PI3K signalling while concurrently promoting IFN-γ release in a TIM-3-dependent manner. This dual effect highlights the context-dependent role of TIM-3 in modulating NK cell effector functions. Mechanistically, galectin-9 also engages CD44 to suppress the proliferation of NK cells, a pathway that is resistant to conventional TIM-3 blockade [67]. This acidification impairs intracellular pH regulation in NK cells, causing mitochondrial ROS accumulation and ATP depletion. Clinically, CRLM patients with lower intratumoural NK cell numbers have higher recurrence rates, highlighting lactate as a key mediator of immune evasion [66]. Hypoxia exacerbates this state through DRP1 (S616)-mediated mitochondrial fragmentation. This fragmentation disrupts citrate-malate shuttling and redistributes H3K27ac histone marks from cytotoxic gene promoters (PRF1 and GZMB) to tolerance-associated loci (LAG-3 and TIM-3⁺), epigenetically locking NK cells into immunosuppressive states [68, 69].

Therapeutic strategies aimed at targeting this plasticity necessitate integrated interventions across multiple dimensions. PD-1/PD-L1 blockade disrupts immunoinhibitory ligand–receptor interactions, thereby restoring cytotoxic effector functions, as demonstrated in clinical models [53, 70–72]. The inhibition of lactate dehydrogenase reverses mTORC1 suppression, reinstating nuclear factor of activated T cells 1 (NFATc1)/IFN-γ signalling and bioenergetic homeostasis [50, 64]. Epigenetic modulators, such as HDAC inhibitors, prevent T-bet silencing and reactivate NKG2D⁺/NKp30 expression. These modulators synergise with TGF-β receptor antagonists to counteract MSC-mediated suppressive signalling. Crucially, these approaches must take into account the spatial heterogeneity observed in EBV^+^ gastric cancer, where cytotoxic and regulatory NK subsets occupy distinct tumour niches. This may require the development of spatially targeted delivery systems to optimise therapeutic efficacy [64].

This integrative framework defines NK cell functional plasticity as a multidimensional adaptation process. Shifts in the receptor expression profile initiate the tolerance state, energetic modifications stabilise the dysfunctional phenotype and metabolic reprogramming enforces bioenergetic constraints. Overcoming these multilayered barriers demands combination strategies that simultaneously target cell-intrinsic reprogramming drivers, such as TBX21 genetic and mitochondrial function and extrinsic TME signals, including TGF-β, lactate and hypoxia. By doing so, the reinstatement of NK cell-mediated immunosuppression across anatomical and molecular gradients becomes achievable. The epigenetic silencing induced by TGF-β converges with metabolic reprogramming in the TME, as discussed in Section ‘Metabolic plasticity: adaptation versus dysfunction’.

## Metabolic plasticity: adaptation versus dysfunction

The metabolic plasticity of NK cells within the TME presents a paradoxical adaptive mechanism. Metabolic reprogramming driven by the imperative for survival in the face of nutrient deprivation ultimately compromises the cytotoxic effector functions of NK cells. Hypoxia-induced lactate accumulation and acidic pH impose substantial bioenergetic stress. This forces NK cells to prioritise the maintenance of cellular homeostasis over their immune surveillance role, thereby triggering a complex cascade of metabolic rewiring events. In breast cancer TMEs, this dualistic adaptation is clearly manifested. Hypoxia also inhibits the proliferation of NK cells and reduces their cytotoxicity [73]. Elevated levels of 27-hydroxycholesterol, a cholesterol metabolite that inhibits the transcriptional regulation mediated by sterol regulatory element-binding protein (SREBP), disrupt glucose metabolism in tumour-infiltrating NK cells [73–76]. Simultaneously, glucose-deprived NK cells activate compensatory glutamine dependency pathways. This metabolic vulnerability can be exploited by glutamine inhibitors such as CB-839, which selectively deplete the energy of tumour cells while safeguarding the viability of NK cells. The metabolic flexibility of NK cells extends to lipid metabolism as well. In HCC models, cholesterol enrichment enhances the antitumour capacity of NK cells through lipid raft-mediated receptor clustering. In contrast, in sepsis, TGF-β suppresses carnitine palmitoyl transferase 1A (CPT1A)-dependent fatty acid oxidation (FAO), leading to mitochondrial fragmentation and a subsequent loss of cytotoxicity [77, 78].

The interaction between nutrient sensing and effector function in NK cells reveals an intricate network of regulatory mechanisms. Tumour-derived kynurenine activates the aryl hydrocarbon receptor (AHR) pathway, which in turn suppresses glycolysis. Conversely, β-hydroxybutyrate restores mitochondrial efficiency and promotes the expression of granzyme B, demonstrating the metabolite-specific modulation of NK cell activity [79, 80]. These adaptations come with functional trade-offs. Although glutamine dependency enables NK cells to survive in glucose-limited niches in the short term, chronic exposure to 27-hydroxycholesterol induces epigenetic silencing of cytotoxicity genes through SREBP-mediated chromatin remodelling. This results in persistent dysfunction of NK cells even after the resolution of metabolic stress [77, 81]. Similarly, lactate accumulation not only inhibits the mTORC1, thereby blocking the nuclear translocation of NFATc1 and the production of IFN-γ, but also redistributes H3K27ac histone marks from the promoters of cytotoxicity genes to tolerance-associated loci. This energetic modification firmly entrenches the immunosuppressive state of NK cells [50, 64].

Emerging therapeutic strategies aim to target these key metabolic nodes through engineered interventions and modulation of the microenvironment. Supplementation with nicotinamide adenine dinucleotide (NAD^+^) enhances the mitochondrial membrane potential in CAR-NK cells. This improvement in mitochondrial function contributes to better cell persistence and a reduction in relapse rates in acute myeloid leukaemia (AML) patients. This approach is paralleled by preclinical studies that use ketone body supplementation to increase mitochondrial efficiency [82]. Moreover, coculturing NK cells with metformin-pretreated MSCs rescues glycolysis in NK cells by upregulating glucose transporter 1 and hexokinase 2. This effectively counteracts the suppressive effects of adipose-derived MSCs, which secrete TGF-β and PGE2 to downregulate the expression of perforin and granzyme B [50]. These strategies highlight the potential of decoupling the bioenergetics of NK cells from the constraints of the TME. However, significant challenges remain in achieving a balance between metabolic rewiring and the preservation of NK cell function.

Spatiotemporal heterogeneity further complicates the targeting of therapeutic interventions. In hypoxic tumour cores, OXPHOS is suppressed through lactate-mediated inhibition of mTORC1. In contrast, normoxic margins allow residual glycolytic activity in NK cells, creating anatomically distinct functional niches. Single-cell analyses confirm this metabolic zonation. In EBV^+^ gastric cancer, IFN-γ^high^ cytotoxic NK subsets are found mainly in the vascularises tumour margins, whereas lipid-laden immunosuppressive NK cells dominate the hypoxic zones [64]. This metabolic compartmentalisation reflects the dichotomous role of cholesterol. In HCC, cholesterol promotes lipid raft formation and the clustering of activating receptors. However, in atherosclerosis models, excessive cholesterol induces oxysterol cytotoxicity, which exacerbates NK cell dysfunction. Such context-dependent outcomes emphasise the need for precision medicine. AML patients may benefit from NAD^+^-enhanced CAR-NK therapies, while solid tumours may require a combined approach targeting lactate transporters (MCT1/4) and AHR signalling to overcome metabolic immunosuppression.

In summary, the metabolic plasticity of NK cells represents a dynamic equilibrium between adaptive survival and functional exhaustion. Therapeutic interventions must carefully navigate the fine line between restoring the bioenergetic competence of NK cells and avoiding iatrogenic metabolic stress. Future strategies that integrate real-time metabolic imaging with spatially resolved omics techniques hold great promise. These strategies could identify niche-specific vulnerabilities, thereby transforming the metabolic adaptation mechanism, which currently promotes tumour survival, into a viable therapeutic target.

## Harnessing plasticity for immunotherapy

### Receptor engineering: iterative innovation and functional optimisation of CAR-NK cells

Cellular immunotherapy encompasses a wide array of modalities, among which chimeric antigen receptor T (CAR-T) cell therapy has achieved extensive clinical implementation. However, recent research breakthroughs have spotlighted CAR-NK cells as a promising alternative, offering several distinct advantages over CAR-T cells. These advantages include better safety, multiple cytotoxic mechanisms, reduced risk of allogeneic reactions and clinical complete remission [83]. A characteristic feature of CAR-NK technology is gradually overcoming the immunosuppressive hurdles of the solid TME through innovative molecular engineering approaches. These cells are predominantly derived from CD56^−^ human cord blood (CB) NK progenitor cells, with the intracellular domains of most CARs relying on the CD3ζ chain signalling module [84]. First-generation CAR-NK constructs solely contain the CD3ζ signalling domain. In contrast, second- and third-generation designs incorporate costimulatory domains, such as the 4-1BB-CD3ζ chimeric structure, which significantly enhance sustained activation and the antitumour memory response. For example, anti-CD19 CAR-NK cells engineered with CD3ζ exhibit a remarkable increase in cytotoxicity against target cells [84, 85].

**Fig. 3:**
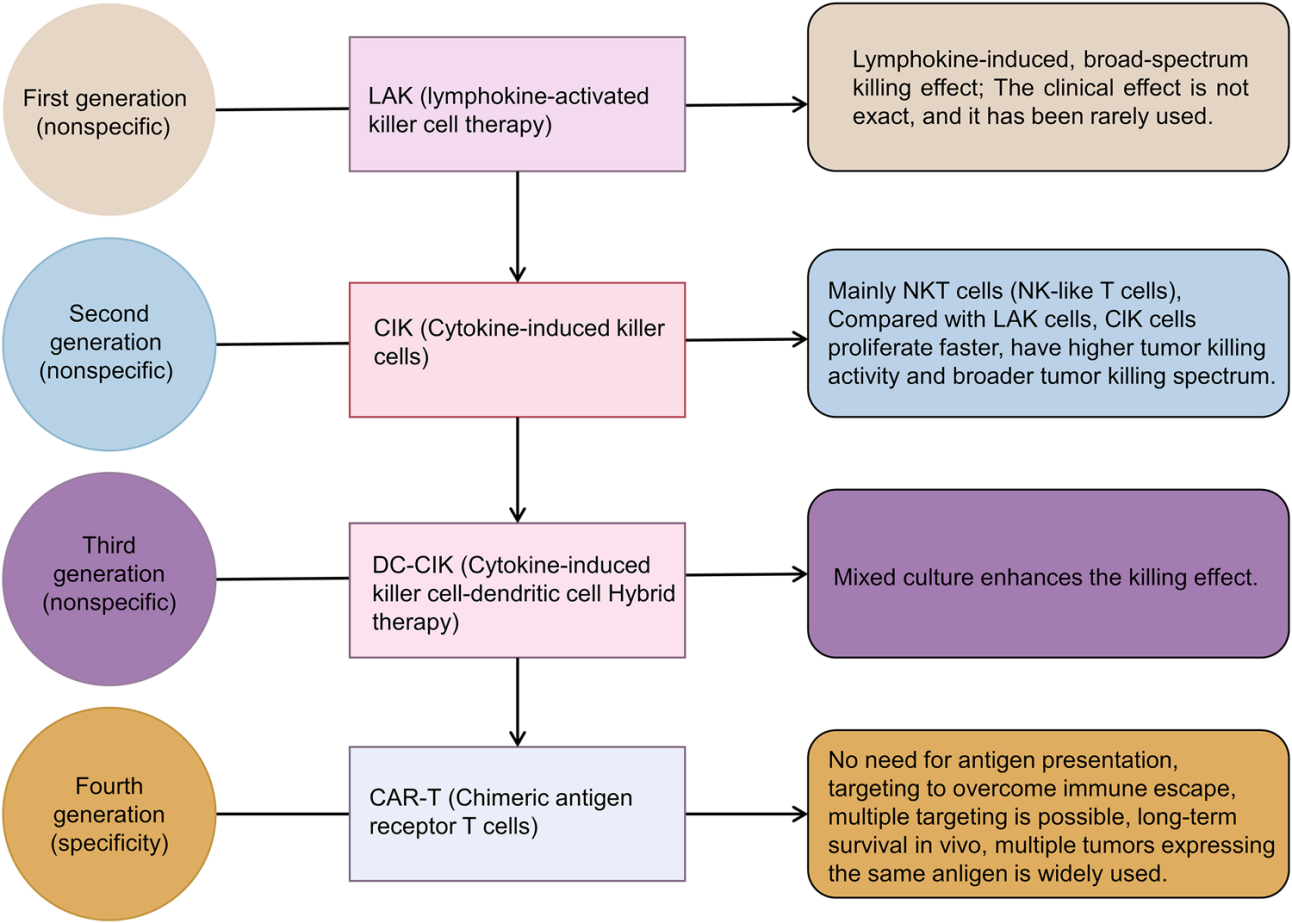
Development of adoptive cell transfer therapy.

The development of CAR-NK molecules has led to the emergence of fourth-generation ‘armed’ CAR systems, which represent a significant departure from earlier designs that relied on native immune receptor domains. These advanced constructs integrate modular molecular payloads, endowing CAR-engineered cells with enhanced functionality and addressing the inherent limitations of conventional immune cell therapies, as depicted in Fig. 3. The iterative evolution of CAR-NK technology across four generations is shown in Fig. 3, highlighting how structural modifications address key clinical challenges. First-generation CAR-NK cells: as shown in the figure, these constructs contain only the CD3ζ signalling domain, enabling basic cytotoxicity but lacking sustained activation—this limits their in vivo persistence, as observed in early CD19-targeted CAR-NK trials for B-cell malignancies [83]. Second/third-generation CAR-NK cells: the addition of costimulatory domains (e.g. 4-1BB-CD3ζ in the second generation, dual costimulatory domains in the third generation) resolves this issue. For example, 4-1BB signalling enhances antitumour memory responses, leading to prolonged CAR-NK survival and 66.7% response rates in diffuse large B-cell lymphoma [86]; fourth-generation ‘armed’ CAR-NK cells: the chart emphasises two key innovations: (1) integration of cytokine genes (e.g. IL-15) to support autonomous persistence—CLDN6-targeted CAR-NK cells with IL-15 modules achieve deep tumour infiltration in pancreatic cancer models [87]; (2) logic-gated antigen recognition (e.g. CD19/CD22, EGFR/IL13Rα2) to reduce off-target toxicity, as exemplified by Senti-401 (CEA-targeted), which spares healthy epithelial cells [88]; notably, Fig. 3 also highlights the shift from ‘native receptor-dependent’ to ‘modular payload-dependent’ design, which is critical for overcoming solid TME immunosuppression—for instance, TGF-βRII mutants in fourth-generation CAR-NK cells block TGF-β-mediated inhibition, a major barrier in glioblastoma [89]; this evolutionary timeline (Fig. 3) underscores how each generation of CAR-NK cells addresses the limitations of the previous generation, providing a framework for current clinical strategies targeting NK cell receptor plasticity. These fourth-generation innovations translate to clinical efficacy in patients with haematological malignancies. In B-cell malignant haematological tumours, CD19-targeted CAR-NK cells, when cocultured with B-cell non-Hodgkin lymphoma cells, increase the secretion of cytotoxic factors and enhance killing activity [85, 90]. The allogeneic logic-gated therapy Senti-401 exemplifies this precision, selectively eliminating carcinoembryonic antigen (CEA)^+^ tumour cells while sparing CEA^+^ healthy epithelial cells, as shown in Table 2 [89, 91–98]. An experiment revealed the role of the endonuclease Regnase-1 in the antitumour activity of NK cells. NK cell-specific deletion of Regnase-1 (Reg1^ΔNK^) increased cytolytic activity and interferon-gamma (IFN-γ) production in vitro and increased intratumoural accumulation of Reg1^ΔNK^-NK cells in vivo, reducing tumour growth in an IFN-γ-dependent manner. It was also proposed that targeting Regnase-1 could increase the persistence of active NK cells, which could be utilised in cancer immunotherapy [99].

**Table 2:** CAR-NK clinical trial progress and key result.

Trial	Target	Indication	Key Outcomes	Reference
CAR19/IL-15 NK	CD19	B-cell malignancies	ORR 48.6%, 1-year OS 68%	[90]
CLDN6-CAR-NK	CLDN6	Pancreatic cancer, Glioblastoma	Deep infiltration, long-term residency capability	[89]
CD33-CAR-NK + anti-PD-L1	CD33, PD-1	Acute myeloid leukaemia (AML)	PD-1 knockout enhances anti-PD-L1 response	[91]
Sentsi-401 (Logic-gated)	CEA	Colorectal cancer	Precision targeting avoids cytotoxicity to normal epithelial cells	[93]
(CAR)-NK cells (NK-92/5.28.z)	HER2	Glioblastoma (GB)	Cytokine release syndrome or ICANS did not occur	[94]
anti-CD38 CAR-NK cell	CD38	Multiple myeloma (MM)	Improve treatment strategies for CD38-positive malignancies	[92]
CD19-BBz CAR-NK	CD19	Refractory/relapsed large B-cell lymphoma	The overall response rate (ORR) was 62.5%	[97]
Memory-like NK cells armed with a TCR-like CAR	HLA-A2	Acute myeloid leukaemia (AML)	improves the antitumor response against an otherwise intracellular mutant protein	[98]

Clinical evidence validates the durability of CAR-NK responses. In a study involving 37 CD19^+^ B-cell malignancy patients treated with cord blood-derived CAR19/IL-15 NK cells, the overall response rate reached 48.6% at both Day 30 and Day 100. The 1-year overall survival and progression-free survival rates were 68% and 32%, respectively. Complete remission was associated with increased CAR-NK cell levels and prolonged persistence. In diffuse large B-cell lymphoma, 4-1BB-stimulated CD19 CAR-NK cells achieved a 66.7% response rate, highlighting the therapeutic potential of integrating costimulatory domains [86, 97, 100].

CRISPR-Cas9-based genome editing has emerged as a potent tool for enhancing CAR-NK functionality. By knocking out inhibitory receptors, such as TIGIT and CD96, it synergises with CAR signalling to reverse TME-induced exhaustion [101, 102]. In AML models, PD-1^-^knockdown CD33^-^targeting CAR-NK cells significantly enhance responses to anti-PD-L1 checkpoint inhibitors, highlighting the synthetic lethality manifested between genetic editing and immunotherapy. Translating NKG2D⁺-based activation into CAR designs, Jin et al. developed dual-targeted CD123/NKG2D ligand CAR-T cells that eliminate AML cells and selectively target immunosuppressive subsets in preclinical models. There are also experiments indicating the potential advantages of targeting TIM-3/CD44 or galectin-9 in the treatment of HPV-positive HNSCC [103, 104]. Unlike T cells, NK cells exhibit minimal adverse reactions and lack the risk of cytokine storms; thus, enhancing the localisation of NKG2D⁺ is a promising strategy for improving the safety of cell therapy [105–107].

### Metabolic reprogramming: resolving TME energy restrictions

The metabolic adaptability of NK cells within the TME has become a focal point of research, with interventions aiming to restore their bioenergetic competence. Mitophagy inducers, such as urolithin A, eliminate dysfunctional mitochondria, thereby restoring OXPHOS efficiency and granzyme B trafficking in NK cells. With respect to lactic acid-mediated NK cell apoptosis, the mitochondrial-targeted antioxidant MitoTempo can effectively block this pathway; under acidic conditions (pH 6.4), MitoTempo reduced the apoptosis rate of liver-resident NK cells from 32.9% to 18.1% (*P* = 0.0013), providing new ideas for clinical translation. Combinations of lactate transporter inhibitors (such as AZD3965) or microenvironmental buffers (bicarbonate) may further enhance the therapeutic effect [66]. IDO inhibitors, such as epacadostat, block tryptophan depletion, preserving mTORC1 activity and the proliferative potential of NK cells [108]. Single-cell metabolomics studies have revealed that the lipid peroxidation microenvironment in the TME upregulates carnitine palmitoyl transferase 1A (CPT1A)-dependent FAO, reshaping the metabolic resilience of NK cells. This provides a mechanistic basis for lipid metabolism-targeted combination therapies [109, 110].

### Cytokine engineering: spatiotemporal control of functional polarisation

Cytokine-based strategies capitalise on the crucial role of soluble factors in regulating NK cells. Next-generation cytokine fusion proteins, such as the IL-15/IL-21 heterodimer, promote NK cell expansion and functional polarisation in melanoma and head and neck cancer models when delivered via spatiotemporally controlled hydrogel systems. Integrating membrane-anchored IL-18 expression into CAR-NK platforms results in the establishment of an autocrine STAT3 activation loop, overcoming TGF-β-mediated immunosuppression [111, 112]. Preclinical data from colorectal cancer liver metastasis models have shown that such engineered NK cells can penetrate the fibrotic stroma, induce immunogenic cell death and stimulate systemic antitumour immunity.

### Microenvironment remodelling: multimodal synergistic interventions

A targeted delivery system composed of a cationic liposome-encapsulated TGF-β siRNA combined with galectin-9 inhibitors disrupted adenosine-dependent inhibition by tumour-associated macrophages (TAMs), significantly increasing the tumour infiltration density of CAR-NK cells. In breast cancer models, this strategy enhances both infiltration and killing activity, demonstrating the potential of nanotechnology to reprogramme the immunosuppressive niche [113–115].

Oncolytic herpes simplex virus expressing CXCL10 (oHSV-CXCL10) utilises chemokine gradients to recruit peripheral NK cells while inducing tumour cells to release damage-associated molecular patterns, thereby amplifying ADCC. In triple-negative breast cancer models, this combination achieves more than threefold greater tumour regression than monotherapy does, highlighting the synergistic potential of viroimmunotherapy, as shown in Table 3 [15, 66, 73, 86, 112].

**Table 3:** Immunotherapy strategy.

Strategies	Mechanisms	Key targets	Advantages	Challenges	Reference
CAR-NK cell	The chimeric antigen receptor (CAR) is genetically engineered to target tumour antigens	CD19 (Haematoma), BCMA (Multiple myeloma)	Low CRS risk, allogeneic use	Poor persistence in vivo, insufficient penetration of solid tumours	[86]
Checkpoint blocking (e.g. TIGIT/PD-1)	Blocking inhibitory receptors and restoring NK cell activity	TIGIT, PD-1, TIM-3⁺	Synergistic with T cell therapy	Off-target cytotoxicity, drug resistance	[15]
Metabolic reprogramming (e.g. MCT1 inhibitors)	Reverse lactate-induced NK cell dysfunction	MCT1, LDHA, IDO	Improved metabolic fitness	Tumour metabolic heterogeneity	[66, 73]
Cytokine engineering (e.g. IL-15/IL-21)	Enhanced proliferation and persistence of NK cells	IL-15 receptor, IL-21 receptor	Prolonging survival time in the body	Cytokine storm risk	[112]

## Challenges and future directions

We have compiled a table summarising the three types of plasticity of NK cells and their interactions, highlighting the importance of NK cells in immunotherapy (Table 4). Despite significant progress, multiple challenges remain in maximising the potential of CAR-NK cell therapy. Single-cell sequencing research has mapped the spatiotemporal heterogeneity of NK cells in EBV-related gastric cancer, revealing distinct transcriptomic profiles between hypoxic core-residing TIM-3⁺ exhausted subsets and marginal CD49a⁺ tissue-resident NK populations [12, 48]. These results emphasize the crucial need for spatial transcriptomics to guide precision treatment strategies, as noted in recent reviews. The host immune rejection of allogeneic CAR-NK cells, along with the metabolic limitations within the TME—which are characterised by nutrient deficiency and lactate build-up—restricts cell persistence. This has led to the exploration of combination therapies with antiangiogenic drugs (such as bevacizumab) to improve tumour infiltration. Moreover, owing to the dysfunction of the patient’s immune system, there is a scarcity of autologous NK cells, which further increases the reliance on allogeneic sources. However, the poor in vitro expandability of NK cells still presents a major obstacle to large-scale production [87]. Additionally, the circulation route of CAR-NK cells poses difficulties. Prolonged exposure to peripheral blood reduces its effective contact with tumour lesions, thereby weakening therapeutic effectiveness.

**Table 4:** Three types of plasticity of NK cells.

Dimension	Key mechanisms	Functional consequences	Therapeutic strategies	Relevant phenotypic markers	Tumour	Reference
Phenotypic plasticity	1. Reprogramming: Inhibitory receptors (TIGIT, TIM-3⁺, PD-1) are upregulated, while activating receptors (NKG2D⁺, DNAM-1⁺) are downregulated； 2. Epigenetic silencing: High methylation of the NKG2D⁺ promoter, HDAC-mediated chromatin compaction of DNAM-1⁺； 3. Subgroup heterogeneity: The hypoxic area is enriched with TIM-3⁺-depleted subgroups and the tissue-resident CD49a⁺ subgroups	1. Loss of target recognition ability 2. Impaired formation of immune synapses 3. Persistent functional impairment	1. Receptor engineering: TIGIT/PD-1 blockers 2. Epigenetic intervention: HDAC inhibitors 3. Subgroup targeting: TIM-3⁺ antibody combination therapy	1. Exhaustive type: TIM-3⁺, PD-1⁺, LAG-3⁺ 2. Tissue-resident type: CD49a⁺, CD103⁺, CXCR6⁺ 3. Immunosuppressive type: CD39⁺, CD73⁺, A2AR⁺	1. Colorectal cancer, liver cancer (TIM-3⁺ depleted type) 2. Non-small cell lung cancer (CD39⁺CD73⁺ immunosuppressive type) 3. Glioblastoma (CD49a⁺ tissue-resident type)	[12, 14, 17, 25–47]
Functional plasticity	1. TGF-β/SMAD3 pathway: recruits HDAC to silence toxic genes (TBX21, PRF1); 2. Metabolic stress: lactic acid inhibits the mTORC1/NFATc1 pathway, reducing IFN-γ production; 3. Spatial polarisation: the tumour margin is enriched with IFN-γ⁺ toxic subpopulations, while the hypoxic core is enriched with PD-L1⁺ immunosuppressive subpopulations.	1. Transformation from cytotoxicity to immunosuppression 2. Decreased ADCC function 3. Loss of immune surveillance function	1. Immune checkpoint blockade: Anti-PD-L1 antibody 2. Metabolic reversal: Lactate dehydrogenase inhibitor 3. Spatial targeted delivery: Hypoxia-responsive drug carrier	1. Function exhaustion: CD107a⁺ (decreased particle release), IFN-γlow 2. Regulatory phenotype: CD56brightCD16⁻, IL-10⁺ 3. Angiogenesis-related: VEGF⁺, IL-10⁺	1. Triple-negative breast cancer (mitochondrial dysfunction induced by TGF-β) 2. EBV-related gastric cancer (reduced IFN-γ secretion of TIM-3⁺ NK cells within the tumour) 3. Glioblastoma (secretion of VEGF by CD56brightCD16⁻ cells)	[38, 48–72]
Metabolic plasticity	1. Energy crisis: Lactic acid accumulation inhibits OXPHOS, while AMPK drives glycolysis; 2. Nutritional competition: Tryptophan depletion activates the IDO-AHR pathway, inhibiting mTORC1; 3. Lipid metabolism disorder: Cholesterol enrichment enhances the cytotoxicity of liver cancer NK cells, but oxysterol induces dysfunction. 4. Metabolic memory: The H3K27ac histone mark redistributes from toxic genes to tolerant genes.	1. Mitochondrial function impairment (TOMM20 ↓) 2. Decreased secretion of granzyme B 3. Chronic metabolic adaptation leading to persistent failure	1. Metabolic intervention: NAD⁺ supplementation, IDO inhibitors (Epacadostat) 2. Lipid metabolism targeting: CPT1A agonists/inhibitors (depending on tumour type) 3. Artificial metabolic support: Ketone body to enhance mitochondrial efficiency	1. Mitochondrial dysfunction: TOMM20 is low 2. Lipid accumulation type: Lipid droplet enrichment, CPT1A⁺ (related to fatty acid oxidation) 3. Metabolic stress type: AMPK activation, mTORC1 inhibition	1. Breast cancer (mitochondrial dysfunction induced by TGF-β) 2. EBV-positive gastric cancer (lipid accumulation in hypoxic areas of NK cells) 3. Acute myeloid leukaemia (NAD⁺ supplementation can improve the function of CAR-NK cells)	[50, 64, 73–82]
Cross-dimensional synergy	1. Epigenetic modifications stabilise metabolic and functional defects; 2. Downregulation of receptors and metabolic inhibition form a positive feedback loop (such as TIGIT inhibiting NKG2D⁺ endocytosis); 3. Spatial heterogeneity (hypoxia/normal oxygenation) drives the coordinated dysregulation of phenotype, function and metabolism.	1. Multidimensional paralysis: Failure of the entire chain from recognition to activation to effectuation. 2. Immunosuppressive microenvironment self-reinforcement	1. Combined therapy: CAR-NK+TGF-β inhibitor + metabolic regulator 2. Microenvironment remodelling: targeting CD73⁺ (adenosine signal), oHSV-CXCL10 virus	1. Composite marker: TIM-3⁺TOMM20low (depletion + mitochondrial abnormalities) 2. CD39⁺CD73⁺PD-1⁺ (immunosuppression + functional exhaustion) 3. CD49a⁺Lipid droplet⁺ (tissue residence + metabolic accumulation)	1. Hepatocellular carcinoma (CD155-TIGIT axis activates and simultaneously inhibits glycolysis and cytotoxicity) 2. Non-small cell lung cancer (CD39⁺CD73⁺ NK cells inhibit T cells through the adenosine pathway, while themselves suffer from impaired metabolic constraint function) 3. Glioblastoma (CD49a⁺ resident NK cells undergo metabolic reprogramming due to hypoxia, with increased VEGF secretion and loss of killing function)	[15, 66, 73, 83–115]

Another layer of complexity comes from stromal cell interactions. Adipose-derived MSCs secrete TGF-β and PGE2. These substances work together to induce NK cell dysfunction through receptor downregulation and metabolic reprogramming. Given that TGF-β regulates the phenotype, function and metabolic plasticity of NK cells by inhibiting the mTOR pathway, intervention strategies targeting this axis may become key to overcoming the limitations of the TME. For example, the combined use of TGF-β receptor antagonists (such as SB-431542) and mTOR activators can simultaneously restore the expression of cytotoxic receptors (such as NKG2D), mitochondrial metabolic efficiency and IFN-γ secretion ability of NK cells. This highlights the need for strategies that simultaneously target both tumour cells and immunosuppressive stromal components.

Innovative methods are emerging to address these challenges. The circadian regulation of NK cell metabolism has become a promising approach for timed therapy. Preclinical studies have shown enhanced efficacy by aligning drug delivery with NK cell metabolic peaks, such as the morning glycolysis peak [116–120]. Building on the pH sensitivity of cytokines in the TME, future efforts could focus on developing microenvironment-adaptable IL-2 variants. For instance, the combination of Switch-2 with immune checkpoint inhibitors (e.g. anti-PD-L1) can restore NK cell function while reversing T-cell exhaustion, demonstrating superior antitumour efficacy compared with monotherapy in the B16.SIY model. Additionally, combining IL-2 variants with metabolic modulators (e.g. lactate transporter inhibitors) could further increase their activity by ameliorating the acidic TME, offering a multidimensional solution to overcome NK cell dysfunction in solid tumours [65]. Integrated multiomics analyses (transcriptomic, metabolomic and epigenomic) hold promise for predicting patient-specific NK cell vulnerabilities, enabling the targeted delivery of miR-155 or NKG2D⁺ agonists via nanoparticles. The synergistic combination of NK cell therapy and antiangiogenic agents has already been demonstrated to be effective in renal cell carcinoma, highlighting the potential of multimodal interventions. Emerging technologies such as spatial proteomics and organoid-based TME modelling will deepen our understanding of NK cell plasticity through the replication of tumour niche complexity. However, standardising NK cell sources is still crucial for clinical translation. Currently, batch differences between manually and machine-separated NK cells affect treatment consistency, making robust quality control frameworks essential.

## Conclusion

The multidimensional plasticity of NK cells—including phenotypic, functional and metabolic adaptations—is not only a core feature of their immune surveillance ability but also a key mechanism for tumour immune escape within the TME. Phenotypic plasticity creates a ‘double-hit’ immunosuppressive pattern through dynamic receptor network remodelling (for example, imbalance of the TIGIT-CD155 axis), epigenetic silencing (such as NKG2D⁺ promoter hypermethylation) and microenvironment-driven subpopulation heterogeneity (e.g. TIM-3⁺ exhausted NK cells in hypoxic niches). Functionally, under the influence of TGF-β/SMAD3/HDAC-mediated epigenetic reprogramming, metabolic stress (such as lactate-induced mTORC1 inhibition) and spatial cues (for instance, the accumulation of PD-L1^+^ NK cells in the cores of EBV^+^ gastric cancer), NK cells shift from cytotoxic effectors to immunosuppressive regulators. Metabolic plasticity reveals a survival paradox for NK cells in the TME: while adaptations such as glutamine dependency or lipid metabolism allow NK cells to tolerate TME stress, these reprogramming events often result in reduced cytotoxic capacity.

In response, emerging immunotherapies use multilevel strategies to overcome TME barriers: receptor engineering (such as combining CAR-NK cells with TGF-βRII mutants), metabolic interventions (for example, NAD^+^ supplementation to restore OXPHOS) and microenvironment remodelling (such as the use of CD73⁺ inhibitors to disrupt adenosine signalling). In the future, technological innovations integrating single-cell spatiotemporal omics, circadian rhythm regulation (such as optimising the CRY/BMAL1 pathway) and precision metabolic imaging will drive a paradigm shift in NK cell therapy, evolving from ‘passive adaptation’ to ‘active remodelling’ of the TME. These advancements offer the hope of achieving breakthroughs in personalised immunotherapy by systematically addressing the complex layers of NK cell plasticity in cancer.
